# Resistive index of central retinal artery is a bioimaging biomarker for severity of diabetic retinopathy

**DOI:** 10.1186/s40942-019-0189-4

**Published:** 2019-11-12

**Authors:** Manila Khatri, Sandeep Saxena, Manoj Kumar, Apjit Kaur Chabbra, Shashi K. Bhasker, Ece Isin Akduman, Hang Pham, Levent Akduman

**Affiliations:** 1 Department of Ophthalmology, King George’s Medical University, Lucknow, U.P. 226003 India; 2 Department of Radiodiagnosis, King George’s Medical University, Lucknow, U.P. India; 3 Department of Radiology, Saint Louis University, St. Louis, MO USA; 4 Department of Ophthalmology, Saint Louis University and The Retina Center, Saint Louis University, St. Louis, MO USA

**Keywords:** Bioimaging, Biomarker, Resistive index, Diabetic retinopathy, Central retinal artery, Optical coherence tomography, Retinal nerve fiber layer

## Abstract

**Background:**

The present study was undertaken to assess the resistive index (RI) of central retinal artery (CRA) as a bioimaging biomarker for the severity of diabetic retinopathy (DR), for the first time.

**Methods:**

Eighty-one consecutive patients of type 2 diabetes mellitus between the ages of 40 and 70 years were included in a tertiary care center-based cross sectional study. Severity of retinopathy was assessed according to Early Treatment Diabetic Retinopathy Study (ETDRS) classification: diabetes mellitus with no retinopathy (No DR) (n = 26); non-proliferative diabetic retinopathy (NPDR) (n = 29); and proliferative diabetic retinopathy (PDR) (n = 26). Twenty-six healthy controls of similar age were also included. Resistive index of CRA was studied using color Doppler and gray scale sonography. Central subfield thickness (CST), cube average thickness (CAT), retinal photoreceptor ellipsoid zone (EZ) disruption, and retinal nerve fiber layer (RNFL) thickness were evaluated using spectral domain optical coherence tomography. Sensitivity and specificity were assessed by receiver operating characteristic (ROC) curve.

**Results:**

Mean RI of CRA for the study groups revealed significant increase with severity of diabetic retinopathy (F = 10.24, P < 0.001). The ROC curve analysis showed diagnostic accuracy of RI of CRA (area under curve = 0.841–0.999; sensitivity = 76–100%, specificity = 95.45–100%, P < 0.001) in discriminating controls and patients. Univariate regression analysis revealed significant association between the study groups and RI of CRA (P < 0.001). RI of CRA correlated positively with CST (r = 0.37), CAT (r = 0.45), EZ disruption (r = 0.43) and negatively with RNFL thickness (r = − 0.35) (P < 0.001).

**Conclusions:**

Resistive index of CRA is a reliable bioimaging biomarker for the severity of DR.

## Introduction

Diabetic retinopathy (DR), a leading cause of morbidity and disability, is a sight threatening micro vascular complication of diabetes mellitus. Prevalence of DR varied from 28.8% in persons who had diabetes for < 5 years to 77.8% in persons who had diabetes for 15 or more years [1].

Diabetic retinopathy is a microvascular disorder. In the setting of raised blood glucose levels, various changes occur in microvasculature of retina that lead to retinal structural and functional changes. The inner 6 layers of the retina are supplied by central retinal artery (CRA) [2]. Spectral domain optical coherence tomography (SD-OCT) is a non-invasive, reliable, imaging tool for in vivo cross sectional retinal histology. The second band initially recognized as inner segment-outer segment junction (IS-OS) of photoreceptor has now been established as the ellipsoid zone (EZ) of the photoreceptor [3]. Earlier, a simplified, comprehensive and physician-friendly classification system to grade EZ disruption correlated significantly with severity of DR and decrease in visual acuity [4].

Color Doppler imaging (CDI) and gray scale sonography is a non-invasive imaging technique that enables measurement of blood flow velocity. Reproducible CDI measurements require adequate training [5]. In a previous study, the subjectivity involved in the analysis of the CDI pictures was assessed. Results from the observer who had been doing the CDI examination on the patients were compared with measurements of these pictures taken by an independent reading center. A good concordance between the observer and the reading center was found from this analysis [6, 7].

The peak systolic (PSV), end diastolic (EDV) and mean blood flow velocities over the cardiac cycle are calculated by built in software. Color Doppler imaging determines velocity of moving cells. Resistive index (RI) computed by PSV and EDV reflects vascular resistance peripheral to the measuring location [8]. The present study evaluated the association of RI of CRA with severity of DR, for the first time.

## Methods

Patients with type 2 diabetes mellitus presenting to this tertiary care center (King George’s Medical University, Lucknow India) were included in this cross sectional study. The authors confirm adherence to the tenets of the Declaration of Helsinki. An institutional review board clearance was obtained. A written informed voluntary consent was obtained from all the study subjects. Diagnosis of type 2 diabetes mellitus was made according to American Diabetes Association (ADA) guidelines which include fasting plasma glucose level ≥ 126 mg/dl, 2 h plasma glucose level ≥ 200 mg/dl during an oral glucose tolerance test [9]. Best corrected visual acuity (BCVA) was measured on logarithm of the minimum angle of resolution (log MAR) scale. All the study subjects underwent stereoscopic fundus evaluation using slit lamp biomicroscopy and indirect ophthalmoscopy. Digital fundus photography and fluorescein angiography were done using a Zeiss fundus camera FF 450 Plus with a pixel width of 0.0054 and an image size of 2588 × 1958 (Carl Zeiss Meditec AG 07740 Jena Germany). Patients with any other ocular or systemic diseases affecting the retinal vascular pathology, history of any previous intravitreal injection(s), ophthalmic surgical or laser intervention, patients on vitamin supplements, antioxidants, any medications causing change in blood flow (calcium channel blockers, pentoxifylline, statins, antiplatelet agents and anticoagulants) and patients giving signal strength of less than 5 on OCT due to media haze at any level were excluded. Eighty-one consecutive patients of type 2 diabetes mellitus attending the retina clinic between age 40 years and 70 years, were included. Right eye of all study subjects was included in symmetrical involvement. In asymmetrical involvement, the eye with more severe form of the disease was included. To assess the severity of DR, patients were divided into three groups, according to Early Treatment Retinopathy Study (ETDRS) classification [10, 11]: diabetes mellitus with no retinopathy (No DR) (n = 26); non-proliferative diabetic retinopathy (NPDR) (n = 29); and proliferative diabetic retinopathy (PDR) (n = 26). Healthy control subjects with no diabetes mellitus, presenting for refraction, were also included (n = 26).

Blood samples were collected from all the study subjects by aseptic venipuncture. Serum urea was measured by kinetic enzymatic method with urease and glutamate dehydrogenase. Serum creatinine was measured by modified Jaffe method without deproteinization. Glycated hemoglobin was measured on autoanalyser using standard protocol.

The study subjects underwent macular thickness analysis using the macular cube (512 × 128) scan feature of SD-OCT (Cirrus High Definition OCT; Carl Zeiss Meditec Inc, Dublin, CA, USA). Diabetic macular edema (DME) was assessed in terms of central subfield thickness (CST) and cube average thickness (CAT) [12]. Central subfield thickness was defined as the retinal thickness of the central 1-mm-diameter circle of the ETDRS grid. Cube average thickness was defined as an overall average thickness for the internal limiting membrane-retinal pigment epithelium tissue layer over the entire 6 × 6 mm square scanned area. On horizontal and vertical SD-OCT scans, retinal photoreceptor EZ disruption was graded into three categories [04], Grade 0: Intact photoreceptor EZ; Grade 1: Focal disruption (photoreceptor EZ disruption indicating subfoveal localized involvement); Grade 2: Global disruption (photoreceptor EZ disruption indicating generalized involvement within the macular cube). Grading was performed by two independent observers masked to the status of retinopathy. For statistical analysis, groups were graded as EZ disruption absent (grade 0) and present (grades 1 and 2). Mean retinal nerve fibre layer (RNFL) thickness in micrometers along the whole circle circumference, four quadrants, twelve clock hours, and at 256 A-scan lengths were obtained on SD-OCT.

Color Doppler and gray scale sonography imaging was done by a single skilled operator, using the Philips Affiniti 70G Ultrasound System Vista, CA, USA. Blood flow was studied in CRA. Vascular resistance against blood flow was calculated by the following formula: RI = (PSV − EDV)/PSV where, RI = resistance index, PSV = peak systolic velocity and EDV = end diastolic velocity.

Statistics: Data were summarized as Mean ± SE. Interobserver correlation for EZ disruption was computed using Spearman rank correlation. Groups were compared by one way analysis of variance (ANOVA) and the significance of mean difference between the groups was done by Newman-Keuls test after ascertaining normality by Shapiro–Wilk’s test and homogeneity of variance between groups by Levene’s test. Categorical groups were compared by Chi square (χ^2^) test. Pearson correlation analysis was done to assess association between the study variables. Sensitivity and specificity of RI of CRA was assessed using receiver operating characteristics (ROC) curve analysis. Univariate regression analysis was also performed. A two-tailed (*α *= 2) P < 0.05 was considered statistically significant. Analyses were performed on SPSS software (Windows version 17.0).

## Results

Mean duration of diabetes in years was 7.16 ± 6.23 in No DR, 10.26 ± 5.88 in NPDR, and 13.08 ± 4.59 in PDR, respectively. Table 1 summarizes the results of ANOVA of biochemical and bioimaging parameters. Analysis of variance showed no statistically significant difference in age among the study groups (F = 1.58, P = 0.265). χ^2^ test showed similar sex proportions among the study groups (χ^2^ = 2.20, P = 0.587). However, ANOVA showed significant difference in log MAR BCVA (F = 109.76, p < 0.001), HbA1C levels (F = 55.87, P < 0.001), serum urea (F = 4.31, P = 0.008) and creatinine (F = 46.546, P < 0.001) among the study groups.

**Table 1:** Demographic, clinical, OCT, topographic and color doppler parameter levels (Mean ± SE) of four groups

Variables	Controls (n = 26) (%)	NO DR (n = 26) (%)	NPDR (n = 29) (%)	PDR (n = 26) (%)	F/χ^2^ value	p value
Age (yrs)	60.45 ± 1.43	60 ± 1.54	58.33 ± 2.32	63.89 ± 1.95	1.58	0.265
Sex						
Female	10 (36.4)	11 (40.9)	9 (28.0)	7 (22.7)	2.20	0.587
Male	16 (63.6)	15 (59.1)	20 (72.0)	19 (77.3)		
HbA1c (%)	5.41 ± 0.12	7.94 ± 0.21	8.50 ± 0.295	8.84 ± 0.167	55.87	<0.001
S. urea (mg/dl)	33.278 ± 0.854	38.078 ± 2.154	37.87 ± 0.978	39.84 ± 1.13	4.31	0.008
S. creatinine (mg/dl)	0.98 ± 0.02	1.16 ± 0.03	1.15 ± 0.03	1.64 ± 0.07	46.546	<0.001
VA (LogMAR)	0.089 ± 0.02	0.36 ± 0.14	0.76 ± 0.07	1.16 ± 0.02	109.76	<0.001
CST (μm)	246.91 ± 2.62	267.73 ± 4.37	308.72 ± 22.52	456.91 ± 19.36	37.21	<0.001
CAT (μm)	255.67 ± 1.08	273.44 ± 7.57	301.06 ± 9.37	371.26 ± 6.23	50.675	<0.001
RNFL (μm)	89.38 ± 0.33	85.57 ± 0.89	86.06 ± 0.92	72.35 ± 1.37	61.14	<0.001
EZ						
Disruption absent	26 (100.0)	23 (95.5)	24 (56.0)	0 (0.0)	60.66	<0.001
Disruption present	0 (0.0)	3 (4.5)	5 (44.0)	26 (100.0)		
RI-CRA	0.66 ± 0.01	0.77 ± 0.01	0.79 ± 0.02	0.987 ± 0.09	10.24	<0.001

*NO DR* no diabetic retinopathy, *NPDR* non proliferative diabetic retinopathy, *PDR* proliferative diabetic retinopathy, *BCVA* best corrected visual acuity, *HBA1c* glycosylated haemoglobin, *CST* central subfield thickness, *CAT* cube average thickness, *RNFL* retinal nerve fiber layer, *EZ* ellipsoid zone, *RI-CRA* resistive index of central retinal artery

SD-OCT based bioimaging parameters: CST, CAT, EZ and RNFL thickness were analysed in the study groups. ANOVA revealed significant difference in CST (F = 37.21, P < 0.001), CAT (F = 50.675, P < 0.001) and RNFL thickness (F = 61.14, P < 0.001) with severity of retinopathy. Interobserver correlation for EZ disruption was observed to be r = 0.78 (P = 0.001). χ^2^ test revealed significant increase in grades of EZ disruption with the severity of retinopathy (χ^2^ = 60.66, P < 0.001).

Color Doppler imaging based vascular RI was analyzed in CRA. ANOVA revealed a significant increase in RI of CRA (F = 10.24, P < 0.001) with severity of DR.

**Fig. 1:**
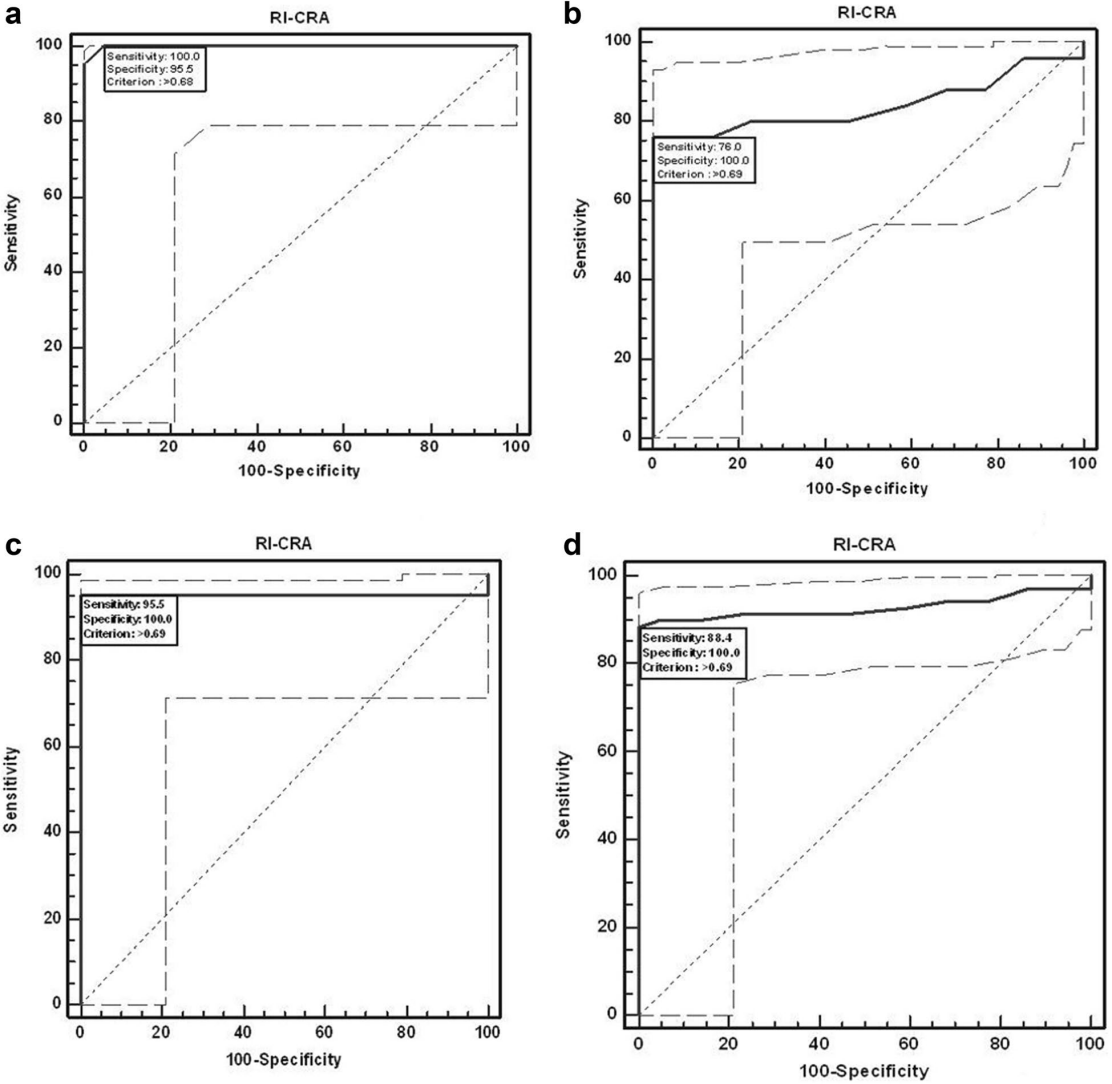
ROC curve analysis shows sensitivity and specificity of RI-CRA to discriminate controls and patients. **a** No diabetic retinopathy, **b** non-proliferative diabetic retinopathy, **c** proliferative diabetic retinopathy, **d** and total patients

**Fig. 2:**
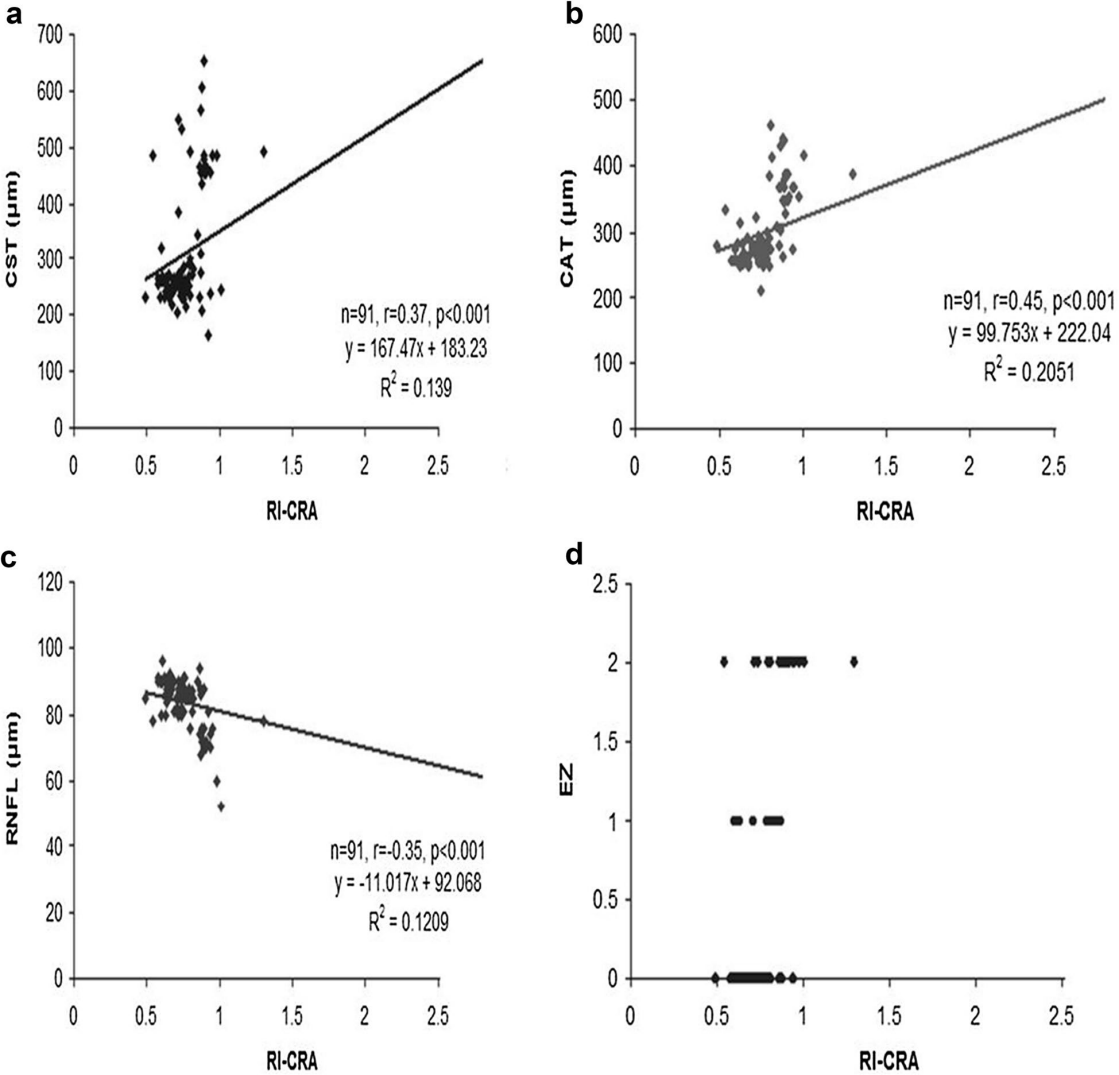
**a** Scatter plot illustrating correlation between resistive index (RI) of central retinal artery (CRA) and central subfield thickness (CST). **b** Scatter plot illustrating correlation between resistive index (RI) of central retinal artery (CRA) and cube average thickness (CAT). **c** Scatter plot illustrating correlation between resistive index (RI) of central retinal artery (CRA) and retinal nerve fibre layer thickness (RNFL). **d** Scatter plot illustrating correlation between resistive index (RI) of central retinal artery (CRA) and ellipsoid zone (EZ) disruption

The ROC curve (Fig. 1a–d) analysis showed diagnostic accuracy of RI of CRA (AUC = 0.841–0.999, P < 0.001) in discriminating controls and patients. RI of CRA had high sensitivity and specificity, at various cut off values, as shown in Table 2. Univariate regression analysis was performed to study the association of RI of CRA with independent variables namely, study groups, age and sex. A significant association was observed between the study groups with RI of CRA (P < 0.001). The association of RI of CRA with age (P = 0.1) and sex (P = 0.3) was not found to be significant. The analyses concluded that RI of CRA is a reliable diagnostic predictor for severity of DR. RI of CRA was found to correlate positively with CST, CAT and grades of EZ disruption (P < 0.001) and negatively with RNFL thickness (P < 0.001) on Pearson correlation analyses (Fig. 2a–d).

**Table 2:** Diagnostic accuracy of RI-CRA to discriminate controls and cases using ROC curve analysis

Group	Criterion (cut off) value	Sensitivity (95% CI	Specificity (95% CI)	+PV	−PV	AUC	Z value	p value
NO DR	> 0.68	100.00 (84.4–100.0)	95.45 (77.1–99.2.)	95.7	100.0	0.999	100.69	< 0.001
NPDR	> 0.69	76.00 (54.9–90.6)	100.00 (84.4–100.0)	100.0	78.6	0.841	5.87	< 0.001
PDR	> 0.69	95.45 (77.1–99.2)	100.00 (84.4–100.0)	100.0	95.7	0.955	13.76	< 0.001
Total	> 0.69	88.41 (78.4–94.8)	100.00 (84.4–100.0)	100.0	73.3	0.928	16.22	< 0.001

+PV, positive predictive value; −PV, negative predictive value; AUC, area under the curve; NA, not applicable, *NO DR* no diabetic retinopathy, *NPDR* non proliferative diabetic retinopathy, *PDR* proliferative diabetic retinopathy

## Discussion

We evaluated the association of RI, a parameter of vascular resistance, in CRA with the severity of DR. Resistive index of CRA was found to correlate significantly with severity of DR. Increase in RI of CRA was found to be associated with increased grades of EZ disruption, increased CST and CAT and decrease in RNFL thickness on SD-OCT. A positive correlation between RI of CRA with Log MAR BCVA was also observed. Area under ROC curve also showed RI of CRA as a significant predictor for severity of DR.

Resistive index has been considered as a marker of vascular resistance. With vascular compliance taken into account, RI was found to increase with increasing resistance [13]. Orbital RI was found to be a useful marker for early diagnosis and follow-up of DR [14]. Resistivity index alteration of the ophthalmic artery and central retinal vein was found to be prevalent among patients with early changes in DR [15].

In patients with early diabetes mellitus with no DR, a significant difference in average venous and arterial velocity was found in small retinal vessels when compared with controls [16]. In another study, pulsatile ocular blood flow was found to be more in patients with no retinopathy as well as retinopathy as compared to controls. Hemodynamic circulation was found to play a causative role in the pathogenesis of DR [17].

Several ocular blood flow studies have been done in patients with DR. Reduced blood flow velocity was found in the CRA of diabetic patients and appeared to become further reduced with the progression of retinopathy [18, 19]. Significant changes in retrobulbar blood flow were found in eyes without and with DR, especially those with retinopathy [20, 21]. Significant circulatory changes were found in the OA, CRA and short posterior ciliary artery in diabetic patients with DR [22].

Basement membrane thickening, pericyte loss [23, 24], increased expression of ICAM-1 [25, 26], oxidative and nitrosative stress [27], rheological changes [28, 29] and decreased capillary perfusion lead to retinal capillary endothelium damage. This results in fluid leakage out of the capillaries resulting in DME, capillary closure and decreased capillary blood flow. These changes lead to decreased blood supply to retina with resultant retinal ischemia and increased vascular endothelial growth factor (VEGF) release [30–32]. Total retinal blood flow was found to be reduced in conjunction with raised levels of aqueous angiogenic cytokines in patients with NPDR [33]. Retinal oximetry studies have shown that increase in retinal venous oxygen saturation is associated with increasing levels of DR. Several studies also found increased retinal arterial oxygen saturation in patients with DR [34].

Retinal blood flow in adjacent capillaries increases due to retinal ischemia. As a result, shear stress in the vessel wall increases. Increase in shear stress in the vessel wall occurs due to increased flow, increased viscosity and capillary closure [35]. Tooke hypothesized that increased glycation and thickening of the basement membrane results in “locking” of the vessel [36]. This tends to increase shear stress since the vessel diameter is unable to change, leading to mechanical injury to the vascular endothelium. Capillary pressure is increased in diabetes mellitus as in the presence of dilated vasculature the systemic blood pressure is more easily transmitted to the microcirculation. Vessel wall of larger vessels of the retinal circulation suffer more circumferential stress damage, as the circumferential stress that is responsible for mechanical damage to the endothelium of the vessel wall is directly proportional to the perfusion pressure and radius and inversely proportional to the thickness of the vessel wall [37]. As a result, the vessel has tendency to dilate. Vessel wall tension resisting distension pressure is inversely proportional to the radius of the vessel, as stated by Laplace law. Vessel has a tendency towards dilatation as the vessel wall tension required to counteract distending pressure is not achieved in a dilated vessel, resulting in subsequent hyperperfusion [38].

In addition, other factors namely, abnormal autoregulation of the retinal circulation [39], increased conductance as an autoregulatory response to retinal ischemia [40], increased activity of nitric oxide synthase, inhibition of calcium influx channel in smooth muscle cells and endothelin-1 resistance also lead to hyperperfusion. As these changes occur in retinal vasculature the resistive index increases. In the present study, we found that an increase in resistive index of CRA correlated with severity of DR.

Central retinal artery supplies the inner 6 layers of retina [2]. Increase in RI of CRA, related to the vascular endothelium damage, was found to correlate significantly with severity of retinopathy as well as an increase in CST, CAT and decrease in RNFL thickness. A significant correlation was also observed with EZ disruption. A study highlighted that deep capillary plexus (DCP) provides around 10–15% of oxygen to photoreceptor inner segment, particularly during dark adaptation [41]. Deep capillary plexus is located on outer side of inner nuclear layer. It receives its branches from CRA. In the setting of systemic hypoxia, blood supply to outer retina through inner retinal vessels becomes even more significant, as choroidal vasculature has no autoregulatory mechanism [42]. Hence, it fails to autoregulate in such a setting [43]. Central retinal artery, through DCP, contributes to vascular supply of outer retina. DCP ischemia, on optical coherence tomography angiography (OCTA), has been found to be associated with disruption of the outer retina, including thinning of the outer nuclear layer and photoreceptor disruption [44].

According to National Institute of Health, biomarker is defined as “a characteristic that is objectively measured and evaluated as an indicator of normal biological processes, pathogenic processes, or pharmacologic responses to a therapeutic intervention” [45]. The ROC curve analysis showed diagnostic accuracy of RI of CRA in discriminating controls and patients and highlighted this vascular parameter as a sensitive and specific biomarker for severity of diabetic retinopathy. Limitation of this study was small sample size. These are preliminary findings that warrant additional work.

To conclude, increase in resistive index of CRA is associated with changes in SD-OCT based parameters and is a simple, reliable, noninvasive, physician-friendly and easy to comprehend bioimaging biomarker for severity of diabetic retinopathy.

## Data Availability

The datasets used and analyzed during the current study are available from the corresponding author on reasonable request.

## Abbreviations

SD-OCT: spectral domain optical coherence tomography
CRA: central retinal artery
DR: diabetic retinopathy
EZ: ellipsoid zone
DME: diabetic macular edema
CST: central subfield thickness
CAT: cube average thickness
RNFL: retinal nerve fiber layer thickness
RI: resistive index

## References

[1] Klein R, Klein BE, Moss SE, Davis MD, DeMets DL (1984). The Wisconsin epidemiologic study of diabetic retinopathy. III. Prevalence and risk of diabetic retinopathy when age at diagnosis is 30 or more years. Arch Ophthalmol..

[2] Blodi FC (1977). Eugene Wolff’s anatomy of the eye and orbit. Arch Ophthalmol.

[3] Spaide RF, Curcio CA (2011). Anatomical correlates to the bands seen in the outer retina by optical coherence tomography: literature review and model. Retina..

[4] Sharma SR, Saxena S, Mishra N, Akduman L, Meyer CH (2014). The association of grades of photoreceptor inner segment-ellipsoid band disruption with severity of retinopathy in type 2 diabetes mellitus. J Case Rep Stud..

[5] Ciulla T, Regillo C, Harris A, Ciulla T (2003). Retina and Optic Nerve Imaging. Book retina and optic nerve imaging.

[6] Stalmans I, Harris A, Fieuws S, Zeyen T, Vanbellinghen V, McCranor L, Siesky B (2009). Color Doppler imaging and ocular pulse amplitude in glaucomatous and healthy eyes. Eur J Ophthalmol.

[7] Stalmans I, Siesky B, Zeyen T, Fieuws S, Harris A (2009). Reproducibility of color Doppler imaging. Graefes Arch Clin Exp Ophthalmol.

[8] Williamson TH, Harris A (1996). Color Doppler ultrasound imaging of the eye and orbit. Surv Ophthalmol.

[9] American Diabetes Association (2015). Diabetes Care.

[10] Early Treatment Diabetic Retinopathy Study Research Group (1991). Early Treatment Diabetic Retinopathy Study design and baseline patient characteristics: ETDRS report number 7. Ophthalmology.

[11] ETDRS Research Group (1991). Grading diabetic retinopathy from stereoscopic color fundus photographs—an extension of the modified Airlie House classification. ETDRS report number 10. Early Treatment Diabetic Retinopathy Study Research Group. Ophthalmology..

[12] Virgili G, Menchini F, Murro V, Peluso E, Rosa F, Casazza G (2011). Optical coherence tomography (OCT) for detection of macular oedema in patients with diabetic retinopathy. Cochrane Database Syst Rev..

[13] Bude RO, Rubin JM (1999). Relationship between the resistive index and vascular compliance and resistance. Radiology.

[14] Basturk T, Albayrak R, Ulas T, Akcay M, Unsal A, Toksoy M, Koc Y (2012). Evaluation of resistive index by color Doppler imaging of orbital arteries in type II diabetes mellitus patients with microalbuminuria. Ren Fail.

[15] Karami M, Janghorbani M, Alireza D, Khaksar K, Kaviani A (2012). Orbital Doppler evaluation of blood flow velocity in patients with diabetic retinopathy. Rev Diabet Stud..

[16] Burgansky-Eliash Z, Barak A, Barash H, Nelson DA, Pupko O, Lowenstein A, Grinvald A, Rubinstein A (2012). Increased retinal blood flow velocity in patients with early diabetes mellitus. Retina..

[17] MacKinnon JR, O’Brien C, Swa K, Aspinall P, Butt Z, Cameron D (1997). Pulsatile ocular blood flow in untreated diabetic retinopathy. Acta Ophthalmol.

[18] MacKinnon JR, McKillop G, O’Brien C, Swa K, Butt Z, Nelson P (2000). Color Doppler imaging of the ocular circulation in diabetic retinopathy. Acta Ophthalmol Scand.

[19] Goebel W, Lieb WE, Ho A, Sergott RC, Farhoumand R, Grehn F (1995). Color Doppler imaging: a new technique to assess orbital blood flow in patients with diabetic retinopathy. Invest Ophthalmol Vis Sci.

[20] Meng N, Liu J, Zhang Y, Ma J, Li H, Qu Y, Meng N (2014). Color Doppler imaging analysis of retrobulbar blood flow velocities in diabetic patients without or with retinopathy: a meta-analysis. J Ultrasound Med.

[21] Sood S, Narang S, Kocchhar S, Sarda S, Aggarwal S, Arya SK (2013). Correlation of progression of diabetic retinopathy with the alterations in retrobulbar circulation. Nepal J Ophthalmol..

[22] Gracner T (2004). Ocular blood flow velocity determined by color Doppler imaging in diabetic retinopathy. Ophthalmologica..

[23] Kuwabara T, Cogan DG (1963). Retinal vascular patterns VI: mural cells of the retinal capillaries. Arch Ophthalmol.

[24] Takahashi K, Brooks RA, Kanse SM, Ghatei MA, Kohner EM (1989). Endothelin I is produced by cultured bovine retinal endothelial cells and endothelin receptors are present on associated pericytes. Diabetes.

[25] Jain A, Saxena S, Khanna VK, Shukla RK, Meyer CH (2013). Status of serum VEGF and ICAM-1 and its association with external limiting membrane and inner segment-outer segment junction disruption in type 2 diabetes mellitus. Mol Vis..

[26] Strozecki P, Kurowski R, Flisinski M, Stefanska A, Odrowaz-Sypniewska G, Manitius J (2013). Advanced glycation end products and arterial stiffness in diabetic and non-diabetic patients with chronic kidney disease. Pol Arch Med Wewn.

[27] Sharma S, Saxena S, Srivastav K, Shukla R, Mishra N, Meyer CH, Kruzliak P, Khanna VK (2015). Nitric oxide levels in diabetic retinopathy and its association with disruption of photoreceptor IS-OS junction and topographic alterations in retinal pigment epithelium. Clin Exp Ophthalmol..

[28] DE McMillan (1983). The effect of diabetes on blood flow properties. Diabetes.

[29] Juhan I, Vague P, Buonocore M, Moulin JP, Jouve R, Vialettes B (1982). Abnormalities of erythrocyte deformability and platelet aggregation in insulin-dependent diabetics corrected by insulin in vivo and in vitro. Lancet.

[30] Patz A (1984). Retinal neovascularization: early contributions of Professor Michaelson and recent observations. Br J Ophthalmol.

[31] Crawford TN, Alfaro DV, Kerrison JB, Jablon EP (2009). Diabetic retinopathy and angiogenesis. Curr Diabetes Rev.

[32] Funatsu H, Yamashita H, Noma H, Shimizu E, Yamashita T, Hori S (2001). Stimulation and inhibition of angiogenesis in diabetic retinopathy. Jpn J Ophthalmol.

[33] Khuu LA, Tayyari F, Sivak JM, Flanagan JG, Singer S, Brent MH, Huang D, Tan O, Hudson C (2017). Aqueous humour concentrations of TGF-β, PLGF and FGF-1 and total retinal blood flow in patients with early non-proliferative diabetic retinopathy. Acta Ophthalmol..

[34] Rilvén S, Torp TL, Grauslund J (2017). Retinal oximetry in patients with ischaemic retinal diseases. Acta Ophthalmol.

[35] Fry DL (1968). Certain histological and chemical responses of the vascular interface to acutely induced mechanical stress in the aorta of the dog. Circ Res.

[36] Tooke JE (1986). Microvascular haemodynamics in diabetes mellitus. Clin Sci..

[37] Burton AC (1954). Relation of structure to function of the tissues of the walls of blood vessels. Physiol Rev.

[38] Hwang TS, Jia Y, Gao SS, Bailey ST, Lauer AK, Flaxel CJ, Wilson DJ, Huang D (2015). Optical coherence tomography angiography features of diabetic retinopathy. Retina..

[39] Grunwald JE, DuPont J, Riva CE (1996). Retinal haemodynamics in patients with early diabetes mellitus. Br J Ophthalmol.

[40] Grunwald JE, Riva CE, Martin DB, Quint AR, Epstein PA (1987). Effect of an insulin-induced decrease in blood glucose on the human diabetic retinal circulation. Ophthalmology.

[41] Birol G, Wang S, Budzynski E, Wangsa-Wirawan ND, Linsenmeier RA (2007). Oxygen distribution and consumption in the macaque retina. Am J Physiol Heart Circ Physiol..

[42] Delaey C, Van De Voorde J (2000). Regulatory mechanisms in the retinal and choroidal circulation. Ophthalmic Res.

[43] Yi J, Liu W, Chen S, Backman V, Sheibani N, Sorenson CM, Fawzi AA, Linsenmeier RA, Zhang HF (2015). Visible light optical coherence tomography measures retinal oxygen metabolic response to systemic oxygenation. Light Sci Appl..

[44] Scarinci F, Jampol LM, Linsenmeier RA, Fawzi AA (2015). Association of diabetic macular non-perfusion with outer retinal disruption on optical coherence tomography. JAMA Ophthalmol..

[45] Strimbu K, Tavel JA (2010). What are Biomarkers?. Curr Opin HIV AIDS..

